# A Pilot Study of Cancer-Induced Bone Pain Using Validated Owner Questionnaires, Serum N-Telopeptide Concentration, Kinetic Analysis, and PET/CT

**DOI:** 10.3389/fvets.2021.637195

**Published:** 2021-12-16

**Authors:** Brian K. Flesner, Bryan T. Torres, Kyle D. Hutcheson, Hansjörg Rindt, Amy R. Zalcman, Charles A. Maitz

**Affiliations:** Department of Veterinary Medicine and Surgery, College of Veterinary Medicine, University of Missouri, Columbia, MO, United States

**Keywords:** osteosarcoma, cancer-induced bone pain, comparative oncology, cancer pain, PET imaging

## Abstract

Cancer-induced bone pain, despite its frequency and severity, is a poorly understood phenomenon in people and animals. Despite excitement regarding translational osteosarcoma studies, there is a lack of attention toward examining cancer pain in dogs. In this pilot study, we used a multimodal pain assessment methodology to evaluate pain relief after therapeutic intervention in dogs with primary bone cancer. We hypothesized that intervention would cause objective evidence of pain relief. Evaluations of 8 dogs with primary bone cancer included ^18^F-FDG PET/CT scans, kinetic analysis, validated owner questionnaires (Canine Brief Pain Inventory, canine BPI), and serum N-telopeptide (NTx) concentration. Dogs were routinely staged and had ^18^F-FDG PET/CT scans prior to treatment with day 0, 7, 14, and 28 canine BPI, serum NTx, orthopedic exam, and kinetic analysis. Dogs treated with zoledronate and radiation underwent day 28 ^18^F-FDG PET scans. All clinical trial work was approved by the University of Missouri IACUC. Four dogs underwent amputation (AMP) for their appendicular bone tumors; four received neoadjuvant zoledronate and hypofractionated radiation therapy (ZOL+RT). Canine BPI revealed significant improvements in pain severity and pain interference scores compared to baseline for all dogs. Positive changes in peak vertical force (+16.7%) and vertical impulse (+29.1%) were noted at day 28 in ZOL+RT dogs. Dogs receiving ZOL+RT had a significant (at least 30%) reduction in serum NTx from baseline compared to amputated dogs (*p* = 0.029). SUV_max_ (*p* = 0.11) and intensity (*p* = 0.013) values from PET scans decreased while tumor uniformity (*p* = 0.017) significantly increased in ZOL+RT-treated tumors; gross tumor volume did not change (*p* = 0.78). Owner questionnaires, kinetic analysis, and ^18^F-FDG PET/CT scans showed improved pain relief in dogs receiving ZOL+RT. Serum NTx levels likely do not directly measure pain, but rather the degree of systemic osteoclastic activity. Larger, prospective studies are warranted to identify the ideal objective indicator of pain relief; however, use of multiple assessors is presumably best. With improved assessment of pain severity and relief in dogs with cancer, we can better evaluate the efficacy of our interventions. This could directly benefit people with cancer pain, potentially decreasing the amount of subtherapeutic novel drugs entering human clinical trials.

## Introduction

The translational value of naturally occurring animal models of pain is understudied and underutilized (1). Cancer-induced bone pain (CIBP), despite its frequency and severity, is a poorly understood phenomenon in humans and animals. Bone is not only a common metastatic site in people; it is one of the most frequent initiators of cancer pain (2–5). The prevalence of metastatic to bone neoplasia and CIBP in veterinary patients is lacking. However, primary bone tumors in dogs, specifically osteosarcoma (OS), have a 10–50-fold increased incidence compared to their human counterparts (6). The similarities between human and canine OS are striking (7): bimodal age distribution, appendicular location, metastatic rate and route, genomic instability, and resistance to chemotherapy in the macroscopic setting. These parallels are exploited in nationwide collaborative efforts to better understand and treat this aggressive disease in dogs, with the hope of positively affecting outcome in their two-legged friends (8). Despite the significant push of comparative OS models, there is a lack of attention toward using the dog to study CIBP. A recent review of translational pain assessment focused on the gaps in currently used experimental animal models (1). The authors described a “crisis” of both translational and reproducible affect, which has contributed to drug development failure rates of over 90% once reaching human clinical phases. Benefits of using natural animal models (i.e., client-owned dogs) over experimental animal models (i.e., laboratory rodents) include the spontaneity of tumor occurrence, shared environments, long-term survival, and genetic diversity (1).

CIBP is multifactorial, consisting of both background and breakthrough pain (9). While background pain may be a dull ache that increases with disease progression, breakthrough pain is short, unpredictable, and difficult to treat with current analgesics (10). The components of CIBP are at minimum tumorigenic, inflammatory, and neurologic (11). Tumors, whether primary or metastatic, invade normal tissue and often induce osteoclasts to break down normal bone. This results in destruction of distal sensory fibers of cancellous/cortical nerves (12, 13) and a painful, acidic environment associated with osteoclastic resorption (14). Inflammatory molecules, whether released by pro- or antineoplastic cells, incite pain through nociceptive activity. Prostaglandins, endothelins, and nerve growth factor may directly activate sensory neurons or alter neurotransmitter expression (10, 11). The neurologic system responds to this inflammatory and cancerous milieu in complex ways. There is direct stimulation of both periosteal, cortical, and marrow sensory and sympathetic nerves (15). At the dorsal root ganglion (DRG), peripheral sensory nerves (whether nociceptive-specific or wide dynamic range) synapse with ascending neurons in the spinal cord (11). There, CIBP differs from simple inflammatory or neuropathic pain (16). Finally, neurochemicals, including substance P and glutamate, may further contribute to CIBP and excite the nervous system (11).

In human CIBP, the mainstay of therapy is radiation therapy (17). As previously mentioned, the complex nature of CIBP mandates multimodal therapy (10). Multiple studies have evaluated biomarkers to better quantify response of CIBP treated with radiotherapy. In a study of over 1,000 patients with bone metastases, a normalized bone-resorption marker (N-telopeptide, NTx) concentration after bisphosphonate treatment resulted in reduced risks of skeletal related events and death (18). However, a systematic review of clinical biomarkers of analgesic response to radiotherapy for CIBP showed no predictor of analgesic response (19). Because CIBP is not only caused by osteoclastic bone resorption or direct tumor destruction, better indicators of pain response are needed to modulate analgesic care.

In dogs, OS has been used as a model to study CIBP. Validation of subjective pain assessment was performed with a questionnaire based on the human Brief Pain Inventory (BPI) (20). This canine BPI was administered to owners of 100 dogs with bone cancer and reliably measured this comparative tumor model. Additionally, a group pioneering the use of bisphosphonates in dogs with CIBP evaluated the use of pamidronate, radiation therapy, and doxorubicin in canine OS. Subjective pain scores, urine NTx excretion, tumor relative bone mineral density, and pressure platform gait analysis were used to assess pain response (21). This same group also evaluated the expression of nociceptive ligands, including nerve growth factor, endothelin-1, and prostaglandin E2 in canine OS cells (22). Most recently, 13 dogs with OS were compared with control dogs to assess quantitative sensory testing, QST, in dogs receiving stepwise palliative analgesic therapy (23). While this study assessed techniques that evaluate central and peripheral sensitization, not all dogs finished the study, and disease progression may have affected the authors' ability to assess efficacy of the analgesics examined. To our knowledge, the use of a multimodal analgesic assessment involving PET scans in dogs treated with standard of care (surgical vs. non-surgical) has not been implemented. Therefore, we aimed to assess CIBP in dogs with multiple subjective and objective tools, while monitoring response during standard treatment for primary bone cancer.

## Methods

### Trial Design

We designed a single-site, two-arm [arm 1 = amputation (AMP), arm 2 = zoledronate and radiation therapy (ZOL+RT)], pilot study evaluating subjective and objective pain measures in client-owned dogs with malignant primary bone tumors presenting to the University of Missouri Veterinary Health Center (MU-VHC). Client-owned dogs of any age, sex, or breed and weighing >20 kg were eligible for enrollment. All dogs were diagnosed with a primary bone tumor of the appendicular skeleton *via* cytology or histopathology. Informed owner consent was collected under an approved University of Missouri Animal Care and Use Committee protocol (#9336). Dogs needed to have adequate bone marrow function as measured by complete blood count and normal organ function as measured by biochemical profile (ALT <3x ULN and normal creatinine) with the exception of ALP, an enzyme frequently elevated in dogs with OS (24). Treatment with NSAIDs and other analgesics was allowed prior to enrollment and during the trial; all dogs were receiving an NSAID before screening commenced. Dogs were not eligible for the study if they were treated with prior chemotherapy, radiation therapy, or surgery, if dogs had evidence of soft tissue or bone metastasis, or if dogs had significant comorbidities that would limit their expected lifespan. All dogs were screened for evidence of pulmonary neoplasia with thoracic radiographs, and lack of metastatic disease was confirmed on baseline whole-body PET/CT scans.

Dogs received baseline (screening) orthopedic examinations, weight/BMI, validated owner questionnaire (20), serum collection for NTx assays, kinetic analysis, and ^18^F-FDG PET/CT scans. Within 7 days of screening, treatment intervention was initiated. Treatments were standardized; however, dogs were not randomized. Ethically, it is inappropriate for veterinarians to mandate amputation for dogs deemed as poor amputation candidates. Client owners were given the option, based on orthopedic soundness, to pursue AMP [complete forequarter (scapulothoracic disarticulation) or hindquarter (femoroacetabular disarticulation)] or ZOL+RT for their dog. Dogs that received AMP had their malignant tumors confirmed by histopathology and could receive adjuvant chemotherapy, based on tumor confirmation and tumor grade. All ZOL+RT dogs received neoadjuvant 0.1 mg/kg zoledronic acid (Mylan Institutional LLC *via* McKesson, NDC 67457-390-54) 24 h prior to their first fraction of radiation therapy. All irradiated dogs received a total of 4 weekly fractions of 8 Gy (32 Gy total central axis dose) from a Siemens ONCOR Impression Plus linear accelerator (Siemens, Munich, Germany).

After starting treatment, dogs followed a weekly assessment of their pain control. This included a 7-, 14-, and 28-day orthopedic exam, owner questionnaire, kinetic analysis, and serum collection for NTx assay. At day 28, a repeat ^18^F-FDG PET/CT scan was performed for ZOL+RT dogs. Standard treatment and follow-up were continued in all dogs after cessation of the trial.

### Validated Pain Questionnaire

Client owners completed the validated canine Brief Pain Inventory (canine BPI) questionnaire, totaling 11 questions, at each visit: baseline, days 7, 14, and 28. The canine BPI tool can be found here: https://www.vet.upenn.edu/research/clinical-trials-vcic/our-services/pennchart/cbpi-tool. Treatment response assessments (positive responder or non-responder) were determined by assignment of canine BPI scores. Briefly, the canine BPI system involves assignment of scores ranging from 0 to 10 on the basis of the degree to which pain appears to interfere with 6 daily activities (Pain Interference Score or PIS; 0 = no interference and 10 = complete interference) and perceived pain severity (Pain Severity Score or PSS; 0 = no pain and 10 = severe pain). For this study, the mean PSS and PIS scores obtained for all dogs at each examination period were compared between treatments (AMP vs. ZOL+RT) and testing time points.

### Biomarker—Serum N-Telopeptide

Whole blood was collected *via* jugular venipuncture at each timepoint as previously described. Whole blood (10 ml) was allowed to clot at room temperature for ~30–45 min. The sample was centrifuged at 2,000 × g for 15 min at room temperature to separate the serum. Leaving the clot undisturbed, serum was removed and placed in polypropylene cryovials. Samples were frozen within 1 h of collection in liquid nitrogen and stored at −80°C until the N-telopeptide assay was performed. Serum NTx concentrations were measured using a commercially available ELISA, Osteomark^®^ NTX (Alere Scarborough, Inc., Scarborough, ME, USA). Values were expressed as normalized nanomolar (nM) bone collagen equivalents (BCE).

### Kinetic Analysis

Kinetic data were obtained at days 0 (baseline), 7, 14, and 28 by use of a pressure sensitive walkway (PSW) system (HR Walkway 4 VersaTek System, Tekscan Inc., South Boston, MA, USA). All dogs were walked on a leash by the same handler on the PSW in an isolated laboratory. Each dog was walked at a velocity of 0.9–1.2 m/s and an acceleration of ±0.5 m/s^2^. The PSW was calibrated according to the manufacturer's specifications, and the vertical ground reaction force (GRF) data obtained from the PSW were reported and analyzed by use of designated software (I scan 5.23, Tekscan Inc, South Boston, MA, USA). Before data acquisition, each dog was weighed on an electronic scale and walked across the walkway a minimum of 3–5 times to allow habituation, or acclimation, to the environment, the PSW, handler, and leash. At least 10 trials were recorded for each dog, and data from the first 5 valid trials were analyzed. A valid trial included a straightforward walk without stopping, hesitating, trotting, or pacing; no overt head movement during the trial; and maintenance of a constant speed on the PSW within the defined velocity and acceleration ranges. For comparison, all values were reported as a percentage of body weight (%BW).

### ^18^F-FDG PET Scans

Dogs underwent baseline ^18^F-FDG PET/CT imaging with a Celesteion PET/CT system (Canon Medical Systems, Tustin, CA, USA) under general anesthesia. Dogs were administered a mean of 3.99 mCi (2.83–4.58 mCi) ^18^F-FDG IV 1 h prior to initiation of scan. Whole-body PET was performed, immediately followed by whole-body CT. Iodinated contrast (Omnipaque 350 at a dosage of 2 ml/kg) was administered, and 30-s and 3-min post-contrast infusion images were acquired. Image assessment was performed by a board-certified radiologist (AZ) using MIM Software Inc. (Cleveland, OH, USA). Tumor SUV_Max_, count intensity, and count uniformity were measured by region-of-interest (ROI) analysis using a combination of RayStation and 3D Slicer (Raysearch Labs, Stockholm, Sweden) (25). SUV, or standard uptake value, is a measure of isotope uptake over dose of ^18^F-FDG administered; it indicates metabolic activity of a specific tissue. Count intensity is a measure of the total counts of a tissue on the PET scan and is not normalized to dose administered. GTV, or gross tumor volume, is measured from the concurrent CT scan and allows one to measure tumor size. Tumor count uniformity is a measure of the metabolic (or other radioisotope) count rate heterogeneity among voxels within a tissue. For neoplastic lesions, the entire volume was contoured on MIM, and the whole tumor volume was evaluated. SUV_Max_ was calculated as the highest point of activity within the tumor volume.

### Statistical Evaluation

This was a pilot trial to evaluate subjective and objective measures of pain. Intra- and inter-treatment arm comparisons were made for dogs receiving non-surgical therapy (ZOL+RT) or surgery (AMP). Data were collected at baseline and 7, 14, and 28 days. For canine BPI, the mean PSS and PIS scores obtained for all dogs at each examination period were compared with an analysis of variance and multiple comparisons were performed with Holm–Sidak's multiple-comparison test (**Figure 2**). For N-telopeptide (NTx) concentrations, data were obtained for all dogs at each examination period. For comparison, a response to therapy was defined by changes from baseline measurements. These values were compared between and within treatment days with an analysis of variance, and multiple comparisons were performed with Holm–Sidak's multiple-comparison test (**Figure 3**). For GRF, only data from dogs in the ZOL+RT arm were available for comparison as response to therapy was defined by changes from baseline measurements. These values were compared between treatment days with an analysis of variance, and multiple comparisons were performed with Holm–Sidak's multiple comparisons test (**Figure 4**). ^18^F-FDG PET scan values were recorded; comparisons were performed between pre- and post-therapy values with a *t*-test, and clinical response to therapy was graphically depicted by changes from baseline measurements (**Figure 5**). All statistical comparisons were performed with the use of GraphPad Prism 6.0 h software (GraphPad Software, San Diego, CA, USA); normality testing was performed for all data, and all comparisons were two-sided with statistical significance set at *p* < 0.05.

## Results

### Recruitment

**Figure 1:**
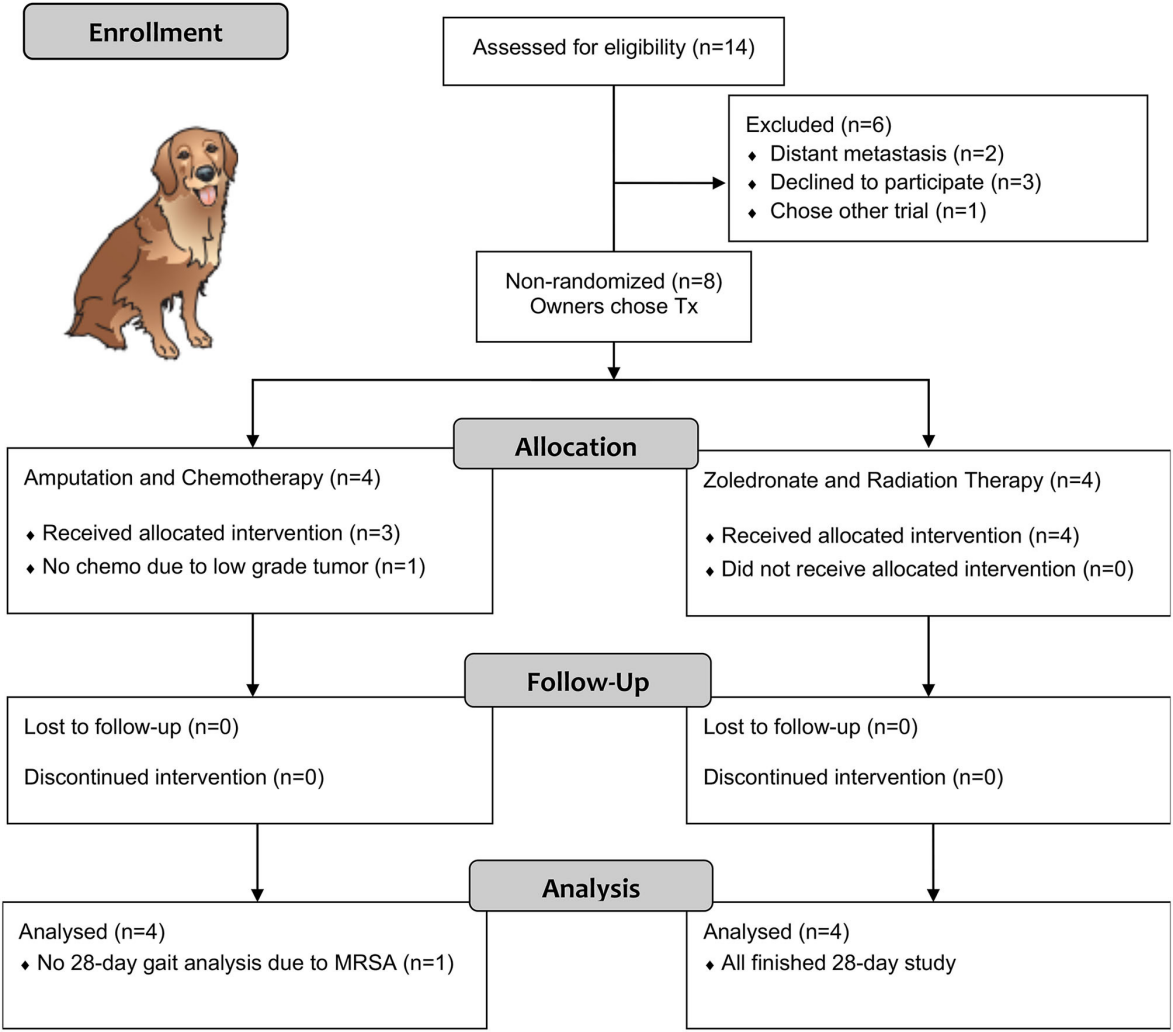
CONSORT diagram of cases in our pilot study.

Fourteen dogs with spontaneously occurring primary bone tumors were screened for the pilot study. Six screened dogs were not enrolled due to distant metastasis or client owner decision to not pursue the trial (Figure 1, CONSORT diagram). Therefore, 8 dogs were enrolled from July to October 2018. Four were enrolled in the AMP arm, and four were enrolled in the ZOL+RT arm. All dogs finished the 28-day study and were then followed according to their clinical treatment protocol. Dog breeds included Golden Retriever (*n* = 2), mixed breed (*n* = 2), Mastiff, Bullmastiff, Labrador Retriever, and Irish Wolfhound. The mean age was 9 years (range 5–13 years), and mean weight was 46.7 kg (range 33.5–72.4 kg), similar to previous publications (7). Tumor locations included the distal radius (*n* = 3), tibia (*n* = 2), proximal humerus (*n* = 2), and distal femur (*n* = 1). Three patients had increased ALP values, a known negative prognostic indicator in dogs (26). ALP values in the rest of the dogs were within reference intervals; ALP was not performed on one dog at screening (only renal panel performed), but ALP was normal on subsequent serum chemistries. An orthopedic examination was performed by a board-certified orthopedic surgeon at the initiation of each visit. During evaluation of study dogs, none of the patients enrolled developed novel sources of musculoskeletal disease (other than previously noted orthopedic or CIBP at baseline).

### Validated Pain Questionnaire

**Figure 2:**
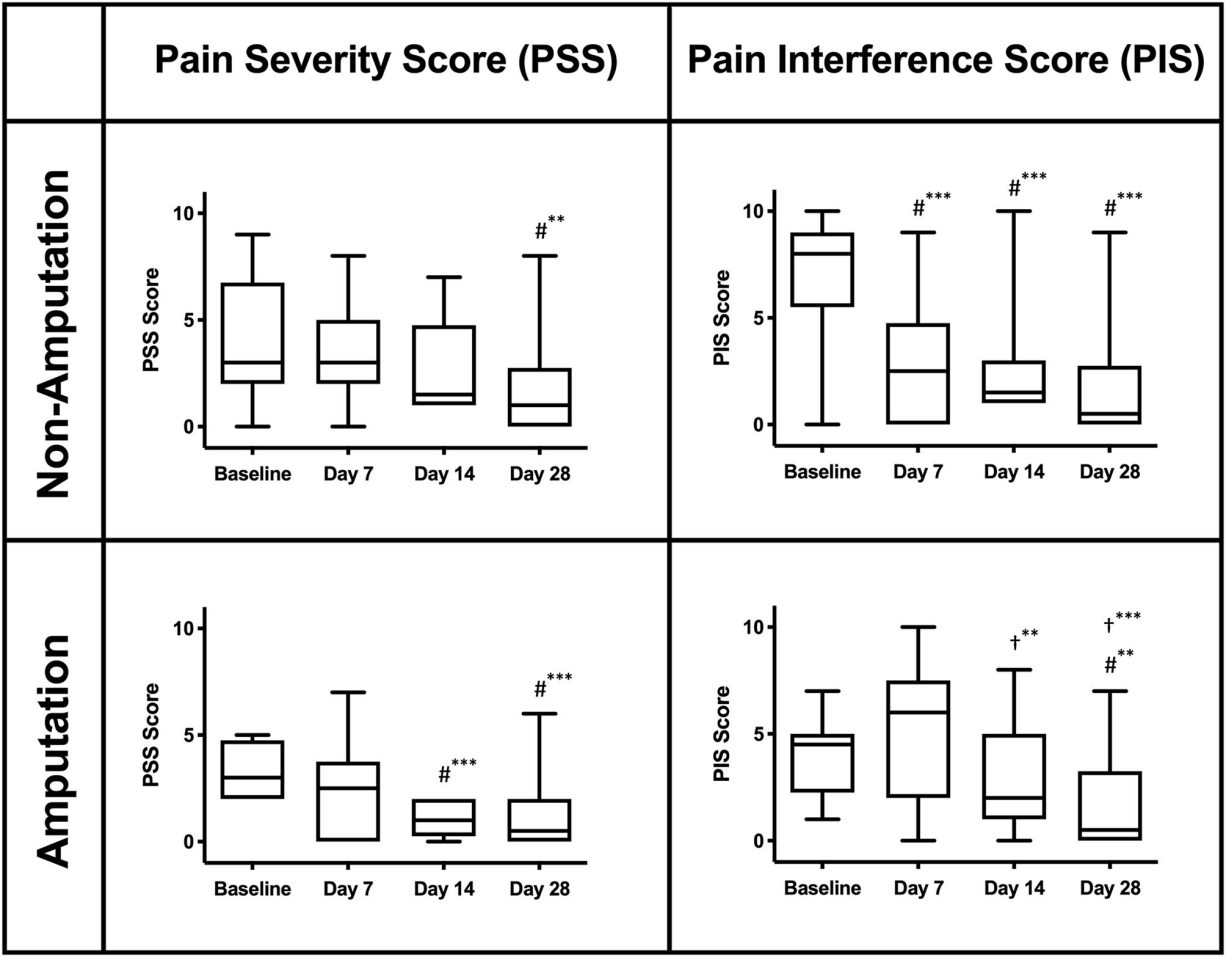
Canine Brief Pain Inventory scores (PSS and PIS) for the affected limb in dogs with and without amputation. A reduction in score indicates a clinical improvement. **(#)** denotes a significant difference compared to baseline. (†) denotes a significant difference compared to day 7. ^**^Significant difference (*p* < 0.05). ^***^Significant difference (*p* < 0.001).

The canine BPI scores [Pain Severity Scores (PSS) and Pain Interference Scores (PIS)] were reported and compared between treatment and time points for dogs with and without an amputation as part of their therapy. The mean (±SD) for PSS were 4.4 (±2.7), 3.4 (±2.5), 2.8 (±2.2), and 1.8 (±2.3) for ZOL+RT dogs and 3.2 (±1.2), 2.3 (±2.1), 1.1 (±0.8), and 1.2 (±1.6) for AMP dogs at days 0, 7, 14, and 28, respectively. The mean (±SD) for PIS were 7.5 (±2.5), 2.9 (±2.7), 2.3 (±2.2), and 1.8 (±2.6) for ZOL+RT dogs and 4.0 (±1.7), 5.1 (±3.4), 2.9 (±2.5), and 1.7 (±2.4) for AMP dogs at days 0, 7, 14, and 28, respectively. There were no significant inter-treatment differences for PSS and PIS on baseline and days 7, 14, and 28. There were significant intra-treatment differences found for PSS and PIS scores compared to baseline for both arms (AMP and ZOL+RT, Figure 2). In all cases, a significant improvement in pain relief, as indicated by a reduction in score, was found for PSS and PIS scores as compared to baseline.

### Biomarker—Serum N-Telopeptide

**Figure 3:**
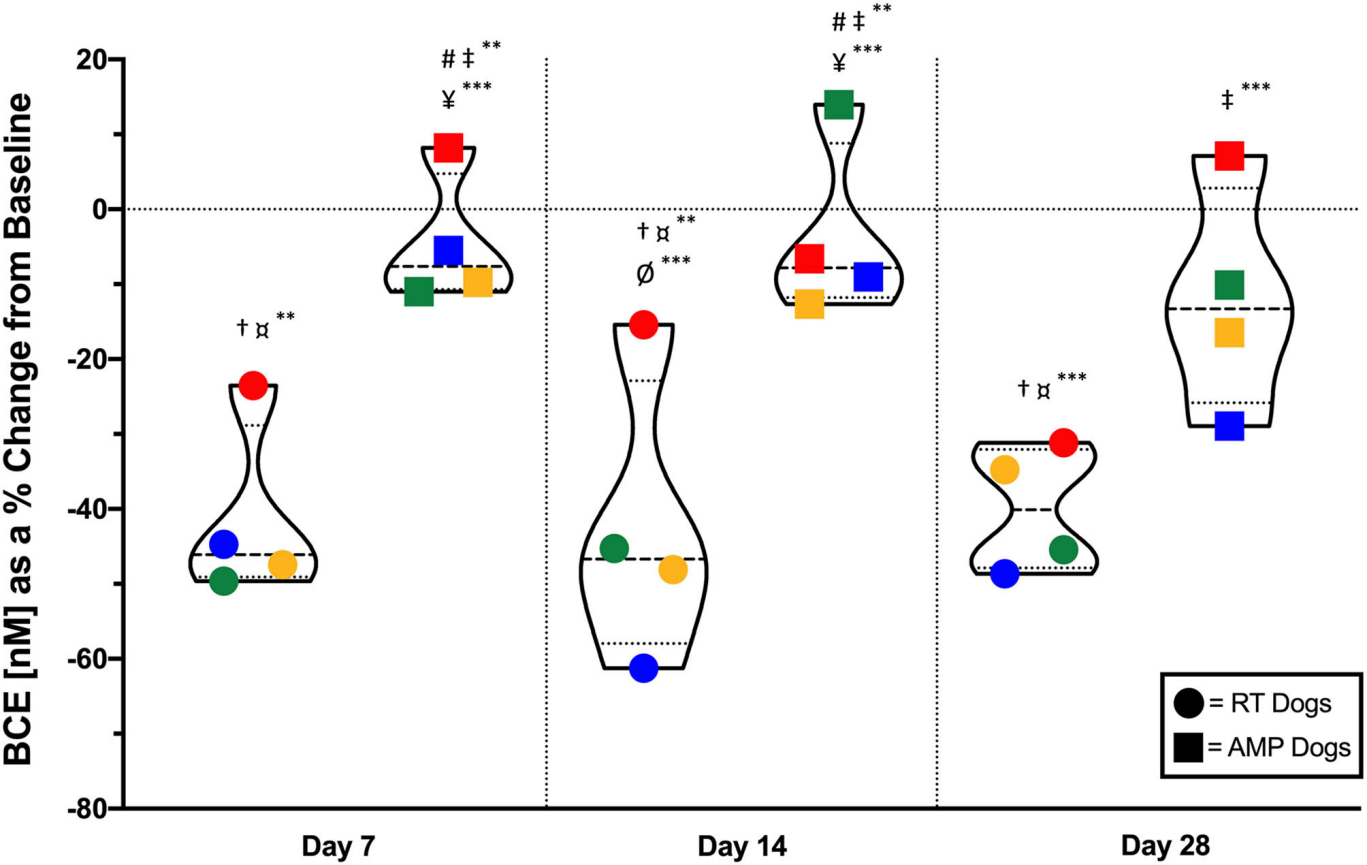
Violin plot of serum N-telopeptide (NTx) concentration, or bone collagen equivalent (BCE), as a percent change from baseline in dogs receiving either zoledronate and RT or amputation for appendicular primary bone tumors. Dogs receiving zoledronate and RT had a 30% or greater percentage decrease in BCE by day 28 of the study; no dogs in the amputation arm had a decrease of 30% (*p* = 0.029). Dogs are grouped by treatment modality (zoledronate and RT, circles; amputation, squares). Individual dogs can be tracked by color in each group. Means and standard deviation are noted by thicker hash mark and thinner hash mark within the violins, respectively. ^#^Significantly different from RT Dogs on Day 7. ^†^Significantly different from AMP Dogs on Day 7. ^‡^Significantly different from RT Dogs on Day 14. ^¤^Significantly different from AMP Dogs on Day 14. ^¥^Significantly different from RT Dogs on Day 28. ^Ø^Significantly different from AMP Dogs on Day 28. ^**^Significant difference (*p* < 0.01). ^***^Significant difference (*p* < 0.05).

N-Telopeptide (NTx) concentration is a direct biomarker of bone turnover, where osteoclasts degrade collagen and release NTx into circulation. Circulating NTx is also referred to as BCE, measured in nM/L. ZOL+RT dogs had a significant (at least 30%) reduction in serum NTx (BCE) from baseline compared to AMP dogs, *p* = 0.029 (Figure 3); significant differences between treatment arms and days are also noted in Figure 3. The mean (±SD) serum NTx (BCE) concentrations were 37.9 (±6.1), 22.7 (±8.3), 22.4 (+/10.9), and 22.8 (±5.6) for ZOL+RT dogs and 3.9 (±12.0), 32.3 (±11.7), 32.9 (±12.9), and 30.7 (±13.1) for AMP dogs at days 0, 7, 14, and 28, respectively. Comparing the two groups, the mean 28-day serum NTx of ZOL+RT dogs (22.8 BCE) was significantly lower than that of AMP dogs (30.7 BCE, *p* < 0.05). All dogs receiving ZOL+RT had a 30% or greater reduction in BCE by day 28; no dogs in the AMP arm had a decrease of 30%, with only one dog reaching >20% reduction in BCE at day 28.

### Kinetic Analysis

**Figure 4:**
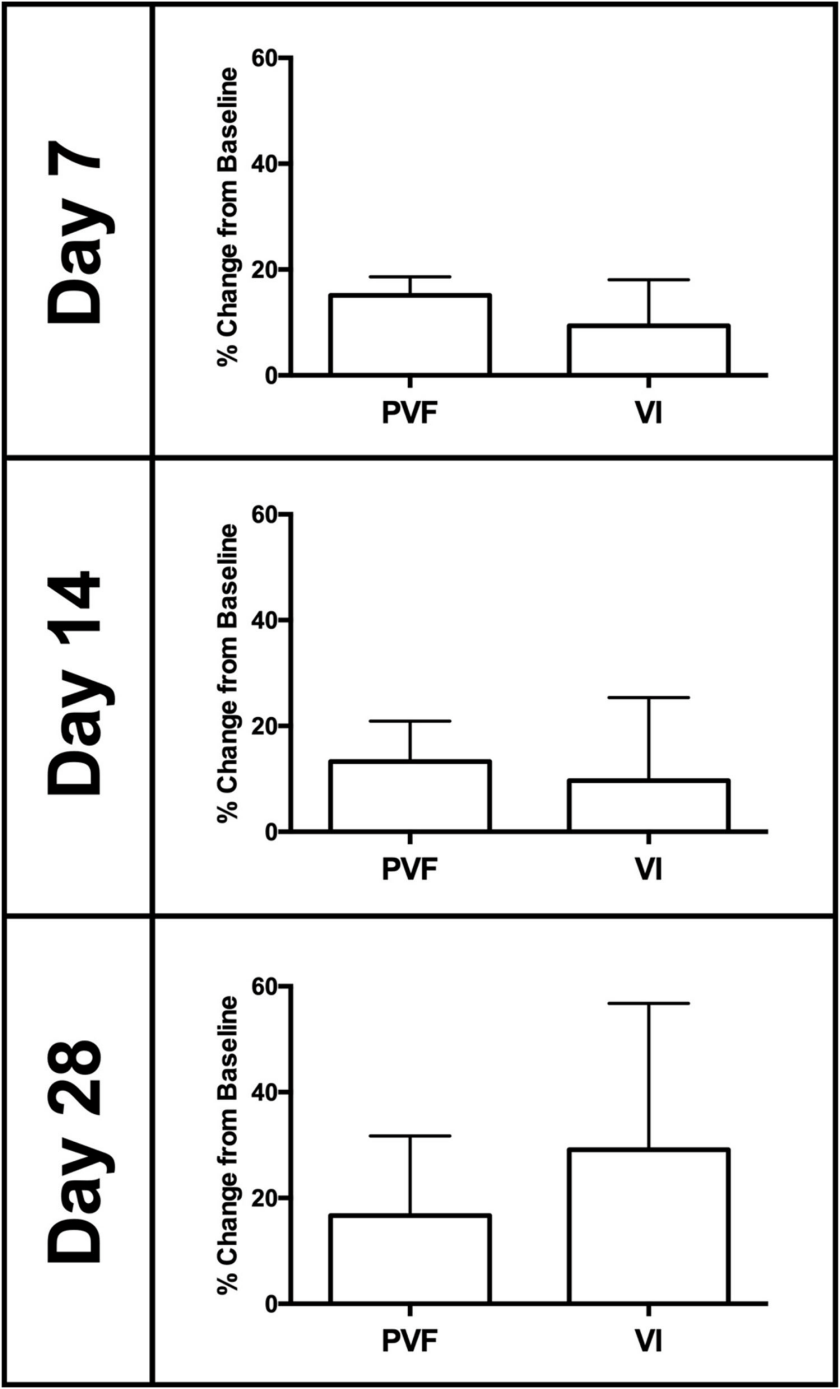
Mean (±SD) ground reaction force (GRF) data (peak vertical force (PVF) and vertical impulse (VI)) presented as a percentage change from baseline (normalized as a percentage of body weight). Data represent the affected limb of dogs with non-surgical treatment (i.e., ZOL+RT). Overall, improvements in GRF measurements (PVF and VI) showed improved weight bearing in the affected limb as compared to baseline measurements, following therapy.

The vertical GRF variables (PVF and VI) from the affected limb in dogs that received ZOL+RT were collected and reported as a percentage change from baseline (Figure 4). For peak vertical force (PVF), the mean percentage change (+/–SD) from baseline was +15.1% (±3.5), +13.3% (±7.7), and +16.7% (±15.0) on days 7, 14, and 28, respectively. For vertical impulse (VI), the mean percentage change (±SD) from baseline was +9.4% (±8.7), +9.7% (±15.7), and +29.1% (±27.6) on days 7, 14, and 28, respectively. Data (PVF and VI) were not available for 2 dogs on day 28. Additionally, comparable GRF data from the affected limb of dogs that received an amputation as a part of therapy were not available for comparison. Reduced pain and improved weight bearing following therapy are indicated by a positive percentage change from baseline. Overall, positive improvements in GRF measurements (PVF and VI) were noted on each testing day as compared to baseline measurements. However, there were no statistically significant differences in these improvements between testing days for PVF or VI.

### ^18^F-FDG PET Scans

**Figure 5:**
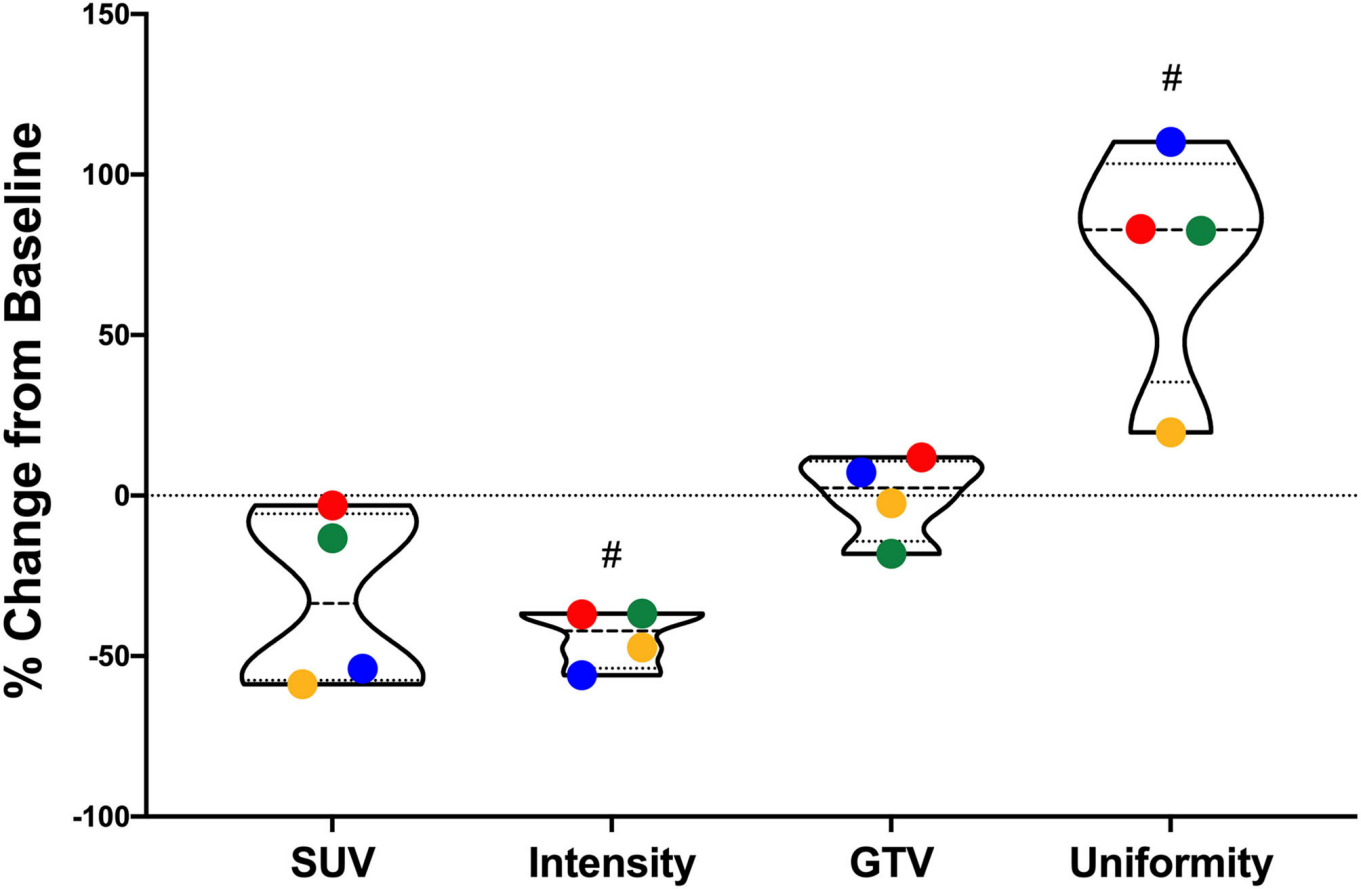
^18^F-FDG PET scan values from dogs receiving zoledronate and coarse fraction radiation therapy for their primary bone tumors. Percent changes from baseline for standard uptake value (SUV), count intensity, gross tumor volume (GTV), and count uniformity are shown. Maximum intensity significantly decreased in all irradiated dogs (*p* = 0.013); maximum SUV decreased, but not significantly (*p* = 0.11). GTV was not statistically different pre- and post-therapy. Tumors became more uniform after therapy (*p* = 0.017). ^#^Significant difference between pre- and posttreatment measurements (*p* < 0.05).

**Figure 6:**
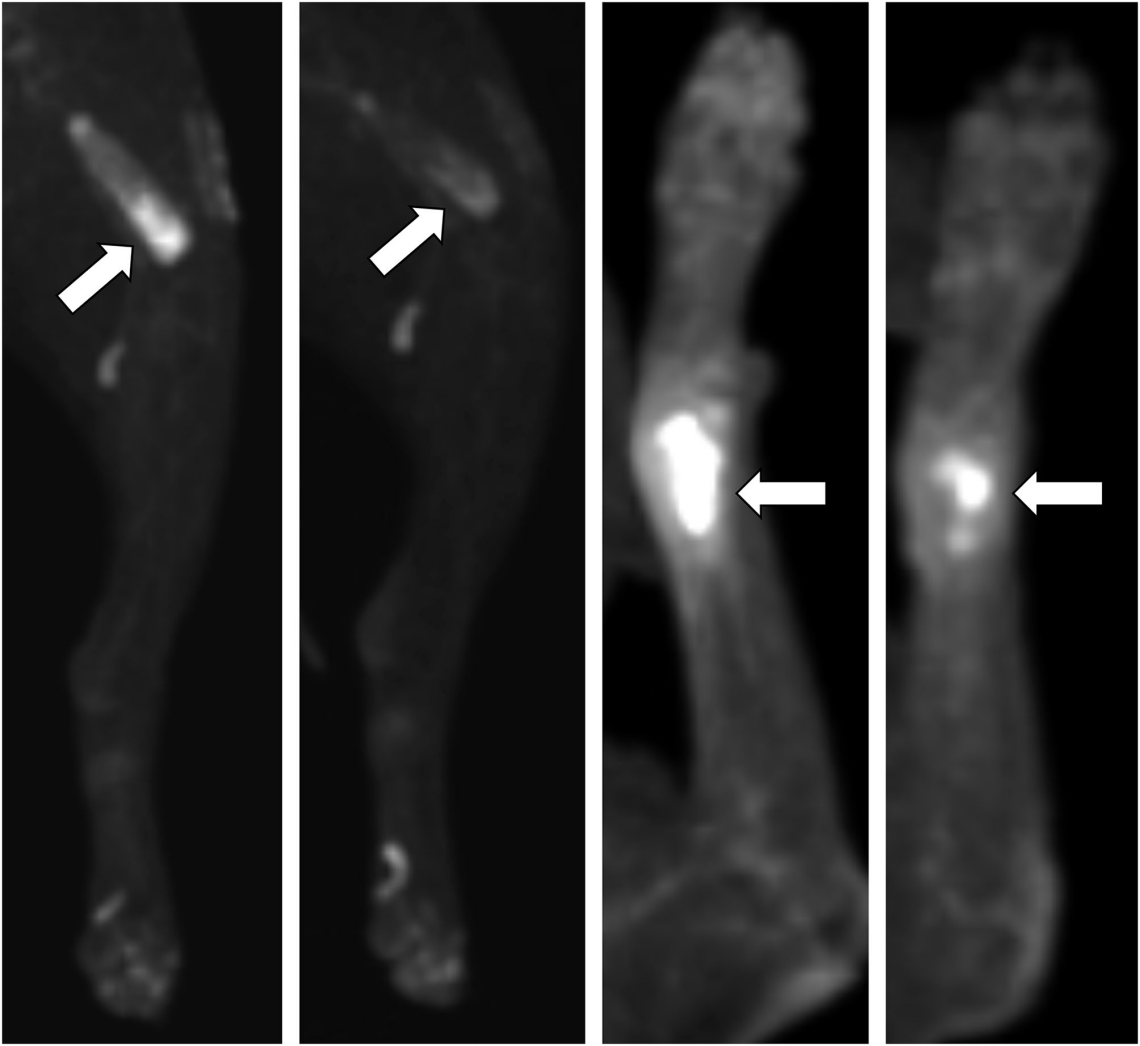
Baseline and day 28 2D images of 18F-FDG PET scans in two dogs with appendicular primary bone tumors receiving ZOL+RT. Both dogs had reductions in standard uptake value (SUV_max_) and tumor count intensity, with no change in gross tumor volume (GTV), and increases in tumor count uniformity. Dog #5 (two left panels) had a distal femur lesion; dog #7 (two right panels) had a distal radius lesion. Primary bone tumors with high intensity 18F-FDG uptake are noted by white arrows, with visual increase in tumor uniformity and decrease in intensity noted on 28-day scans.

All dogs had a screening 18F-FDG PET/CT scan. However, only dogs receiving ZOL+RT had a repeat day 28 PET scan. Figure 5 shows percent changes from baseline for 18F-FDG PET scans. Mean, standard deviation, % change, and **p**-values are provided in Table 1. Maximum intensity significantly decreased in all irradiated dogs (*p* = 0.013); maximum SUV decreased, but not significantly (*p* = 0.11). Gross tumor volume was not statistically different pre- and post-therapy, meaning that tumors did not grow/shrink. Tumors became more uniform after zoledronate and RT (*p* = 0.017). Visual representations of the increase in uniformity and decrease in intensity of two dogs receiving zoledronate and RT are shown in Figure 6.

**Table 1:** 18F-FDG PET variables assessed in dogs receiving zoledronate and radiation therapy (ZOL+RT) for appendicular osteosarcoma.

**PET variable**	**Scan**	**Mean**	**SD**	**% Change**	***p***
Tumor SUV max	Baseline	9.9	0.2	−32.3	0.107
	Post-Tx	6.7	2.7		
Tumor count intensity	Baseline	8501.0	3012.0	−44.3	^*^0.013
	Post-Tx	4729.0	2005.0		
Gross tumor volume	Baseline	43.5	16.6	−0.3	0.781
	Post-Tx	44.4	21.8		
Tumor count uniformity	Baseline	0.0048	0.0019	73.8	^*^0.017
	Post-Tx	0.0078	0.0013		

* *Signifies statistical significance p < 0.05*.

## Discussion

We achieved our goal of assessing pain in dogs with primary bone tumors using a multimodal pain assessment methodology. However, our results do not indicate which objective/subjective marker should play the most significant role. This is in part due to the small sample size of our pilot study resulting in the inability to perform correlative statistical analysis and to the multifactorial causes of cancer pain. A key problem of relying on one pain indicator is shown by serum NTx, which is often touted as a way to assess disease burden and/or pain in animals and people with widespread osteolysis (27, 28). While dogs in our trial receiving zoledronate and radiation therapy had significant decreases (>30%) in serum NTx at 28 days after treatment, dogs with tumors that had been completely removed (i.e., amputation) did not have corresponding reductions in serum NTx. A false conclusion would be that dogs receiving amputation for their primary bone tumors do not have resolution of their pain. More likely, especially when validated owner questionnaires are simultaneously evaluated over 4 weeks, those dogs' pain did improve after recovering from amputation (albeit delayed due to postoperative recovery time). A potential cause for static serum NTx is systemic bone remodeling to account for altered weight bearing on three limbs. This is not to say that the other methods we analyzed are perfect for monitoring pain in dogs receiving more than one therapy, as kinetic analysis and PET imaging require tumors to remain “on” the subject for valid follow-up analysis.

Pain scales are used widely in human medicine and often focus on degree of pain noted by a visual face scale and depend on the patient's or patient guardian's assessment of his/her pain (29, 30). Because questioning veterinary patients about their pain is impossible, veterinarians often rate their patients' pain retrospectively *via* owner questionnaires (31) or descriptive/numerical scales (32). Due to interobserver variability and owner bias, multidimensional pain scales (33), posture/facial expression [specifically in cats (34, 35)], and quantitative sensory testing (36, 37) have more recently been used. However, almost all of these pain assessments are used in induced-pain models or in veterinary patients undergoing surgery. While the aforementioned canine BPI (20) study was validated in dogs with primary bone tumors, comprehensive pain studies in naturally occurring, chronic pain diseases are lacking.

Kinetic analysis in dogs is an objective metric that is widely utilized as an indirect measure of pain in dogs. It is frequently used to assess treatment efficacy in chronic painful conditions such as osteoarthritis. In this study, positive improvements in GRF data were demonstrated in the ZOL+RT dogs at each time point when compared to baseline measurements. However, these improvements were static with no statistically significant changes between testing days. Overall, a >13% improvement was seen in PVF for all time points and >9% was seen in VI. These findings demonstrate that kinetic data can be collected in dogs with CIBP and can provide valuable objective assessments. However, these data are gleaned from a limited number of dogs in this pilot study and the population of dogs available for post-treatment kinetic testing was diminutive as compared to what would be expected in a larger clinical trial. It is possible that a larger population or longer follow-up testing would have detected differences between testing time points. Additionally, the impact of sources of variability on this smaller population size is not known. Kinetic data collection in dogs, especially those with chronic painful conditions, can be influenced by factors including habituation, handler variability, and extended physical exertion. Efforts were made to reduce the impact of these sources of viability in this study population. Habituation to the testing facility and laboratory environment is critically important. Previous studies have demonstrated that adequate habituation can improve kinetic data collected in dogs by reducing data variability (38). In this study, animals were allowed to habituate to the testing environment for a minimum of 3–5 min prior to data collection. Prolonged physical exertion can also impact kinetic data collection, and previous studies have demonstrated that kinetic analysis in dogs with lameness secondary to osteoarthritis can be exacerbated by exercise (39). The impact of physical exertion on trials collected later in each testing period in this study population is unknown. However, all efforts were made to facilitate timely and efficient data collection. Finally, data variability has been shown to be impacted by different handlers and can result in up to 7% of data variance (40). To address this, all dogs were walked by the same handler at all-time points.

Our study is the first to include PET imaging in the assessment of dogs with CIBP. In human medicine, several groups have evaluated ^18^F-FDG PET scans in patients receiving RT for painful bone metastases (41–43). Those studies have shown that SUV_max_ could be used to predict the improvement in pain for people receiving palliative radiation for their metastases, as well as pre-RT pain severity. One study of 74 patients diagnosed with non-small-cell lung cancer bone metastases showed that higher pre-RT SUV_max_ resulted in worse progression-free and event-free survivals (43). The relationship of ^18^F-FDG PET SUV_max_ and pain or response to therapy is likely due to glucose metabolism's relationship to growth rate and biologic aggressiveness of a tumor (43). In another chronic pain translational model, cats with osteoarthritis-associated pain underwent ^18^F-FDG PET scans (44); in 7 cats with OA, significant differences in brain metabolism were noted compared to normal cats. While not directly assessing pain, another group of veterinary researchers evaluated ^18^F-FDG PET scans in dogs with OS (45). This group found that dogs with treatment-naïve OS with ^18^F-FDG PET scan SUV_max_ values ≥7.4 had shorter survival times than dogs with SUV_max_ < 7.4. However, this study was retrospective and dogs were allowed to have either surgical (amputation vs. limb-spare) or stereotactic radiation, and follow-up PET scans were not performed. While our study sample size is small, patients were prospectively enrolled, treatments were standardized, and our pilot study findings warrant further investigation into the role of PET scans in assessing pain in dogs with cancer.

A weakness of many canine OS studies is lack of definitive diagnosis. In fact, because of confirmation of our cases with either cytology or histopathology by a board-certified pathologist, one of the dogs in our study was diagnosed with primary bone chondrosarcoma, instead of osteosarcoma. The dog was included in the amputation arm but did not receive chemotherapy due to the low-grade nature of its tumor. Historical pain studies in dogs with aggressive bone neoplasms have not uniformly confirmed a diagnosis of OS (20, 23), instead relying on radiographic findings, signalment, and tumor location. The lack of definitive diagnosis could result in inclusion of primary bone tumors of non-osteoid lineage, potentially affecting outcomes such as progression-free survival and overall survival. Future prospective studies should always confirm diagnosis of OS, *via* either ALP-positive cytology or histopathology, to remove non-osteoid primary bone tumors' skewing of results.

## Conclusion

Validated owner questionnaires (canine BPI), kinetic analysis, and ^18^F-FDG PET scans showed improved pain relief in dogs with appendicular primary bone tumors receiving ZOL+RT. Serum NTx levels likely do not directly measure pain, but rather the degree of systemic osteoclastic activity. Larger, prospective studies are warranted to identify the ideal objective indicator of pain relief; however, use of multiple assessors is presumably best. Ongoing projects in our oncology/orthopedic research group at the University of Missouri include evaluating novel pain targets in dogs with PET imaging. With improved description and quantification of pain severity and relief in dogs with cancer, we can better evaluate the efficacy of our interventions. Dogs could prove to be a better cancer pain model because of their similar and shared environment with humans, genetic diversity, long-term survival compared to rodents, and naturally occurring (rather than induced) neoplasms. This could directly benefit people with CIBP, potentially decreasing the amount of subtherapeutic novel drugs entering human clinical trials.

## Data Availability Statement

The raw data supporting the conclusions of this article will be made available by the authors, without undue reservation.

## Ethics Statement

The animal study was reviewed and approved by University of Missouri IACUC. Written informed consent was obtained from the owners for the participation of their animals in this study.

## Author Contributions

BF, BT, and KH designed the study. BF ensured all patients met the enrolment criteria and completed the study as scheduled. BF and BT assessed canine BPI. BF and HR performed NTx assays. BT and KH performed orthopedic exams and kinetic analysis. CM and AZ analyzed FDG PET scans. CM and BF were involved in treatment plans. BT performed statistical analysis with assistance from HR and BF. All authors contributed to writing and editing the manuscript and contributed to the study.

## Funding

Intramural funds were used for the clinical trial.

## Conflict of Interest

The authors declare that the research was conducted in the absence of any commercial or financial relationships that could be construed as a potential conflict of interest.

## Publisher's Note

All claims expressed in this article are solely those of the authors and do not necessarily represent those of their affiliated organizations, or those of the publisher, the editors and the reviewers. Any product that may be evaluated in this article, or claim that may be made by its manufacturer, is not guaranteed or endorsed by the publisher.
